# Networks Are Associated With Depression in Patients With Parkinson’s Disease: A Resting-State Imaging Study

**DOI:** 10.3389/fnins.2020.573538

**Published:** 2021-02-09

**Authors:** Haiyan Liao, Sainan Cai, Qin Shen, Jie Fan, Tianyu Wang, Yuheng Zi, Zhenni Mao, Weijun Situ, Jun Liu, Ting Zou, Jinyao Yi, Xiongzhao Zhu, Changlian Tan

**Affiliations:** ^1^Department of Radiology, The Second Xiangya Hospital, Central South University, Changsha, China; ^2^Medical Psychological Center, The Second Xiangya Hospital, Central South University, Changsha, China; ^3^Department of Neurology, The Second Xiangya Hospital, Central South University, Changsha, China

**Keywords:** Parkinson’s disease, depression, independent component analysis, triple-network model, resting state

## Abstract

**Background:**

Disturbance of networks was recently proposed to be associated with the occurrence of depression in Parkinson’s disease (PD). However, the neurobiological mechanism of depression underlying PD remains unclear.

**Objective:**

This study was conducted to investigate whether intra-network and inter-network brain connectivity is differently changed in PD patients with and without depression (PDD and PDND patients, respectively).

**Methods:**

Forty-one PDD patients, 64 PDND patients, and 55 healthy controls (HCs) underwent resting-state functional magnetic resonance imaging (fMRI). The default mode network (DMN), executive control network (ECN), salience network (SN), precuneus network (PCUN), and sensorimotor network (SMN) were extracted using independent component analysis (ICA), and then the functional connectivity (FC) values within and between these networks were measured.

**Results:**

PDD patients exhibited abnormal FC values within the DMN, ECN, SN, PCUN, and SMN. In addition, PDD patients demonstrated decreased connectivity between anterior SN (aSN) and bilateral ECN, between posterior SN (pSN) and dorsal DMN (dDMN), and between PCUN and dDMN/SMN/bilateral ECN. Connectivity within the left hippocampus of dDMN and the right medial superior frontal gyrus of aSN was a significant predictor of depression level in PD patients.

**Conclusions:**

Aberrant intra- and inter-network FC is involved in several important hubs in the large-scale networks, which can be a biomarker for distinguishing PDD from PDND.

## Introduction

Parkinson’s disease (PD) is the second most prevalent neurodegenerative disease (Mason et al., 2014); it is characterized by a combination of cardinal motor features and non-motor symptoms (NMS), including different prominent psychiatric disturbances (McLaughlin et al., 2014). A progressive degeneration of the nigrostriatal dopaminergic projections leads to classical motor symptoms in PD (Davie, 2008). Depression is one of the most common NMSs in PD, and almost half of PD patients have mild to major depressive symptoms (Kalia and Lang, 2015). Unfortunately, rarely have studies analyzed the circuit-level degeneration in PD with depression (PDD).

The recently proposed “triple-network” model emphasized the cooperation among networks. The triple-network model includes the default mode network (DMN), executive control network (ECN), and salience network (SN) (Menon, 2011). They are the three most important intrinsic networks of the human brain activation. DMN controls internal thought and autobiographical memory (Buckner et al., 2008). ECN is involved in multiple cognitive processes, such as attention, language, and memory (Lerman et al., 2014). SN has both abundant structural connectivity and functional connectivity (FC) with many brain regions of the DMN and ECN (Averbeck and Seo, 2008; Craig, 2009), and it is well known for its functions in processing salience information (Craig, 2009) and regulating balance between different large-scale networks (Menon, 2011) such as DMN and ECN. Abnormalities in the triple-network model have been observed across several psychiatric disorders including PDD (Manoliu et al., 2014; Wang et al., 2014; Fan et al., 2017; Weng et al., 2019). However, these network abnormalities were found in PD patients from a small cohort. The precuneus network (PCUN) hub consists of the precuneus, posterior inferior parietal lobule, middle cingulate cortex, and dorsal angular gyri. PCUN responds to a wide range of cognitive processes, including self-related processing (Lou et al., 2004); reflective, awareness, and conscious information processing (Vogt and Laureys, 2005); episodic memory (Dörfel et al., 2009); and visuospatial processing (Wenderoth et al., 2005). The sensorimotor network (SMN) includes somatosensory (e.g., postcentral gyrus) and motor (e.g., precentral gyrus) regions and extends to the supplementary motor areas. However, the connectivity within these specific networks has not been documented in PDD patients.

Resting-state functional magnetic resonance imaging (RS-fMRI) is a novel, non-invasive approach without performing any specific task. Previous RS-fMRI studies in depressed PD patients using the amplitude of low-frequency fluctuation (ALFF) and regional homogeneity (ReHo) analysis have demonstrated alterations in regional brain activity in the prefrontal–limbic system, the basal ganglia (BGN), and the DMN (Wen et al., 2013; Luo et al., 2014; Sheng et al., 2014; Hu et al., 2015a). Studies with FC analysis have observed FC disruption in the emotional, limbic, motor, and DMNs in depressed PD patients (Hu et al., 2015b; Huang et al., 2015; Liang et al., 2016). However, the networks do not function independently, and investigation of the connectivity within one specific network may not be enough or may be somewhat unjustified. A study using independent component (IC) analysis (ICA) confirmed the involvement of BGN, DMN, left frontoparietal network (LFPN), and SN in depression in PD patients, facilitating the development of more detailed and integrative neural models of PDD (Wei et al., 2017). Based on the above studies, we hypothesized that PDD may be related to the damage of specific large-scale neural networks, rather than on the disruption of a single, random, discrete brain region; not only the connectivity within these networks but also the coordination between them can be a prominent feature of PDD.

Alternatively, the ICA is a seed-free approach for detecting connectivity of brain networks. ICA offers an effective means for identification of functional systems within the brain during rest, typically referred to as “resting-state networks” (RSNs) or “intrinsic connectivity networks” (ICNs) (Beckmann et al., 2005; Damoiseaux et al., 2006). The ICA components extracted by enforcing orthogonality spatially represent the brain networks, while the FC correlation values between each network pair reflect interaction. Thus, ICA not only can investigate the FC within one specific network but also can test the FC across the networks. Moreover, using ICA, the subnetworks can be independently extracted. A previous study with a small sample size suggested that ICA is effective to identify the abnormal intrinsic FC within and between large-scale neural networks in depressed PD patients (Wei et al., 2017). Therefore, we proposed that determining neural correlations with ICA could lead to the discovery of biomarkers to improve the diagnosis of PDD.

This study was proposed to use the RS-fMRI and ICA to systematically investigate not only the connectivity within but also the connectivity between DMN, ECN, SN, PCUN, and SMN in PDD patients and PD without depression (PDND) patients. Specifically, we first used the ICA to identify and decompose the networks into independent *z*-maps (reflecting the intrinsic FC of areas within each network, intra-FC), and then we computed the FC correlation values between each network pair representing the intrinsic FC between networks (inter-FC). Second, we would compare the intra-FC and inter-FC data among PDD patients, PDND patients, and healthy controls (HCs).

## Materials and Methods

### Participants

Forty-one PDD, 64 PDND patients, and 55 HCs were recruited with a written informed consent from the Second Xiangya Hospital between December 2015 and February 2020. The study was approved by the Ethical Committee of Medical Research of the Second Xiangya Hospital (2015-533). The criteria for recruited patients were as follows: (1) they met the United Kingdom Parkinson’s Disease Society Brain Bank Criteria (Daniel and Lees, 1993); (2) they were right-handed Han Chinese; (3) they stopped anti-PD or anti-depressant medications for 12 h before imaging and neuropsychological examination; (4) their motor symptoms were evaluated by the Unified Parkinson’s Disease Rating Scale (UPDRS-III) and Hoehn and Yahr (H-Y) scale; (5) they had an H-Y staging score of between 1 and 3; (6) depression was diagnosed with the *Diagnostic and Statistical Manual of Mental Disorders*, Fifth Edition (DSM-V) criteria by an experienced psychiatrist, and patients had a 17-item Hamilton Depression Rating Scale (HDRS-17) score higher than seven (Wang et al., 2018). Afterward, the severity of depression was quantified with the HDRS-17 and the 21-item Beck’s Depression Inventory (BDI); and (7) they had no apparent cognitive impairments, which were determined by the Mini-Mental State Examination (MMSE) score not falling lower than the corresponding education level (Li et al., 2016). An MMSE score >17 for illiterate participants, >20 for grade-school literate participants, and >23 for junior high school and higher education literate participants were defined as normal MMSE scores in our patients. Patients were excluded if they had a history of head injury, stroke, or other neurologic or psychiatric disease; had an abnormal MMSE score; or had any disorders that interfere assessment of the manifestations of PD (Wang et al., 2020). The same exclusion criteria listed above were applied to the control group. Neuropsychological examinations were used to excluded dementia and depression in controls. Five PDD patients, four PDND patients, and three HCs were excluded from the present analyses due to the stringent inclusion and exclusion criteria. The demographic and clinical information is presented in Table 1.

**TABLE 1:** Demographic and clinical characteristics of the participants (mean ± SD).

Variable	HC (*N* = 52)	PDD (*N* = 36)	PDND (*N* = 60)	*P*
Gender (M/F)	23/29	15/21	35/25	0.189^a^
Age (years)	56.212 ± 8.465	56.361 ± 8.319	56.950 ± 9.967	0.903^b^
Education (years)	8.087 ± 3.656	6.694 ± 3.069	7.367 ± 3.361	0.168^b^
Disease duration (years)	NA	2.667 ± 2.588	2.304 ± 1.733	0.415^c^
MMSE	26.673 ± 3.294	26.250 ± 2.882	27.067 ± 2.469	0.401^b^
HDRS-17	2.289 ± 2.615	14.639 ± 4.859	3.283 ± 2.116	<0.001^b^
BDI-21	9.804 ± 9.353	23.000 ± 10.570	8.733 ± 5.862	<0.001^b^
UPDRS-III	NA	19.056 ± 11.810	14.800 ± 9.896	0.061^c^
H-Y	NA	1.639 ± 0.743	1.550 ± 0.642	0.538^c^
REL-GMV	0.408 ± 0.028	0.405 ± 0.026	0.403 ± 0.028	0.686^b^
REL-WMV	0.338 ± 0.021	0.337 ± 0.02	0.335 ± 0.024	0.727^b^
Mean Power_FD	0.096 ± 0.05	0.074 ± 0.037	0.09 ± 0.04	0.078^b^

*Significant *post hoc* two-sample *t*-tests (*p* < 0.05, Bonferroni corrected): DRS-17: PDD *vs.* PDND; PDD *vs.* HC; BDI-21: PDD *vs.* PDND; PDD *vs.* HC. PDD, Parkinson’s disease with depression; PDND, PD patients without depression; HC, healthy control; HDRS-17,17-item Hamilton Depression Rating Scale; BDI-21, 21-item Beck’s Depression Inventory; UPDRS-III, Unified Parkinson’s Disease Rating Scale-motor part III; H-Y, Hoehn and Yahr staging scale; MMSE, Mini-Mental State Examination; REL-GMV, relative gray matter volume; REL-WMV, relative white matter volume; FD, framewise displacement; NA, not applicable. For comparisons of demographics: ^*a*^*p* value for the gender difference was obtained by chi-square test. ^*b*^*p* values were obtained by one-way analysis of variance (ANOVA) tests. ^*c*^*p* values were obtained by two-sample *t*-test.*

### MR Image Acquisition and Data Preprocessing

The procedure and parameters to obtain resting-state fMRI images were previously described (Liao et al., 2020). During the image processing, the first 10 volumes were removed from each participants’ series, slice timing was aligned, and the head motion was corrected. Data from nine participants (five PDD and four PDND) who displayed excessive head motion were excluded from further analyses. The images were then spatially normalized to a standard Montreal Neurological Institute (MNI) template, resampled to the voxel size of 3 mm × 3 mm × 3 mm, and spatially smoothed with a Gaussian kernel (8 mm × 8 mm × 8 mm full width at half maximum).

### Independent Component Analysis and Selection of Networks of Interest

The ICs were analyzed by the Group ICA (the GIFT software^1^). The procedures based on this toolbox included three steps: (1) data reduction, (2) applying ICA algorithm, and (3) back-reconstruction for individual-level components. In this study, 38 ICs were auto-estimated through the minimum description length (MDL) criteria, and Group ICA was performed 20 times. Nine meaningful components were identified as anatomically and functionally classical RSNs *via* visual inspection. They were extracted from all participants, including the ventral DMN (vDMN), dorsal DMN (dDMN), PCUN, two SMNs, right ECN (RECN), left ECN (LECN), anterior SN (aSN), and two posterior SNs (pSN). Seven were related to the “triple-network” model, with three components reflecting the SN (one for the aSN and two for the pSN), two reflecting the DMN (one each for the vDMN and dDMN), and two reflecting the ECN (one each for the LECN and RECN) subnetworks.

### Outcome Measures

The 10 ICs corresponding to the triple networks, PCUN, and SMN were then extracted from all participants. For each subject, each component’s *z*-map and its corresponding temporal cortex (TC) reflected the measures of intra-FC. Linear detrending, despiking, and temporal filtering were conducted for all TCs of the networks of interest before evaluating the inter-FC (Buckner et al., 2008; Luo et al., 2014). We calculated Pearson’s correlation coefficients of the TCs for each network pair among the SN, DMN, central executive network (CEN) subsystems; PCUN; and SMN and then transformed the coefficients into *z*-scores by Fisher’s *z*-transformation. These transformed *z*-scores indexed the inter-FC of each network pair.

### Statistical Analysis

SPSS v19 software was used for data analysis (IBM, United States). The differential brain regions were identified among the three groups using one-way analyses of variance (ANOVAs) and *post hoc* two-sample *t*-test in a pair-wise manner.

To statistically analyze the FC within each selected ICN, it was calculated voxel-wise with participants’ reconstructed spatial maps using one-sample *t*-test SPM12 [*p* < 0.001, false discovery rate (FDR) corrected]. Comparisons of connectivity within each ICN among PDD, PDND, and HC groups were performed using a design model of one-way ANOVA in SPM12, followed by *post hoc* two-sample *t*-test (voxel level, *p* < 0.001, cluster size >10 voxels, corresponding to a corrected *p* < 0.05 as determined by AlphaSim correction). The between-groups differences of connectivity among selected ICNs were assessed using ANOVA, and *post hoc* two-sample *t*-test was used to determine connectivity changes between each pair of the three groups (*p* < 0.05, AlphaSim corrected).

The correlations between the detected connectivity abnormalities and the HDRS as well as the UPDRS-III scores were assessed using Pearson correlation coefficient for the PDD and PDND patients. A *p* < 0.05 was considered significant.

To test whether the intra-connectivity can predict depression level in the PD patients, hierarchical regression analyses were performed on the depression level as indicated by HDRS scores. Five regression models were run in total. Age, gender, educational years, MMSE scores, UPDRS-III scores, and illness duration were entered as predictors in step 1. To evaluate which connectivity could significantly predict depression level and beyond the predictors included in step 1, regression model 1 included connectivity of all four brain regions, which were different between PDD and PDND as predictors in step 2, and a stepwise method was used to determine which predictors to retain in the final model. To evaluate the predictive value of connectivity of each of the four regions, the connectivity of one of the four regions was entered as the only predictor in step 2 for each of the other four regression models.

## Results

### Demographic Information

No significant differences in H-Y staging, UPDRS-III, and PD duration were observed between the PDD and PDND patients, while no significant differences in gender, age, education level, relative gray matter volume (REL-GMV), MMSE scores, relative white matter volume (REL-WMV), and mean FD were observed between the three groups (Table 1). In contrast, significant differences in the HDRS-17 and BDI scores were observed among the three groups (*p* < 0.001).

### Identification of Network of Interests

**FIGURE 1:**
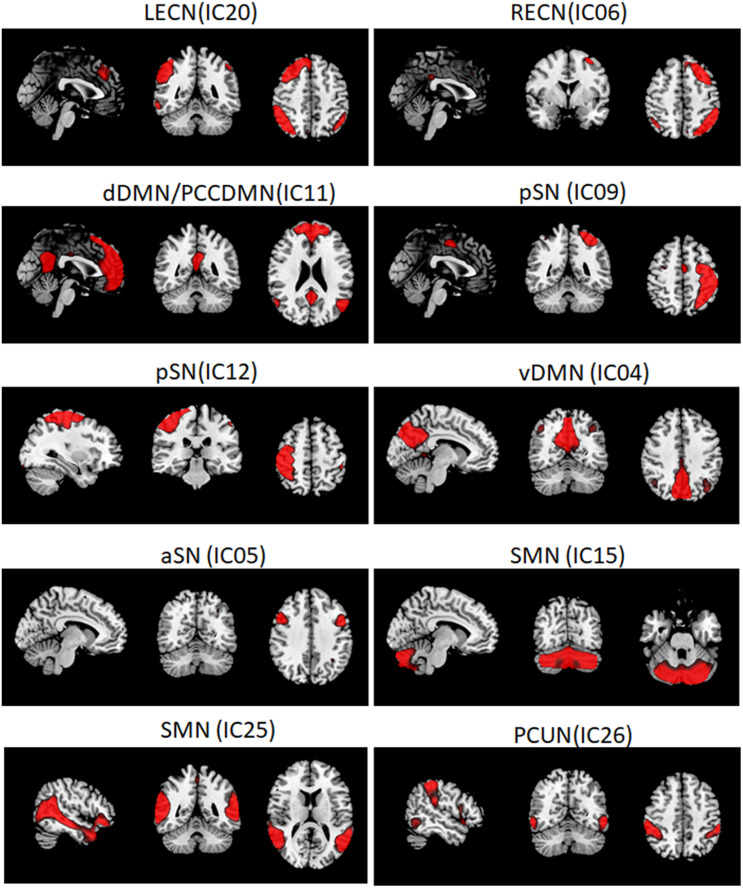
Large-scale networks including the 10 resting-state networks (RSNs) identified by independent component analysis. Left executive control network, LECN (IC20); right executive control network, RECN (IC06); dorsal default mode network, dDMN (IC11); posterior salience network, pSN (IC09/IC12); ventral default mode network, vDMN (IC04); anterior salience network, aSN (IC05); sensorimotor network, SMN (IC15/IC25); precuneus network, PCUN (IC26).

The respective spatial patterns of 10 networks of interests for the three groups (PDD, PDND, and HCs) were revealed by the one-sample *t*-test (Figure 1, FDR corrected, *p* < 0.05), including the LECN (IC20), RECN (IC06), dDMN (IC11), pSN (IC09/12), vDMN (IC04), aSN (IC05), SMN (IC15/IC25), and PCUN (IC26).

### Intra-Network Connectivity Analysis

**FIGURE 2:**
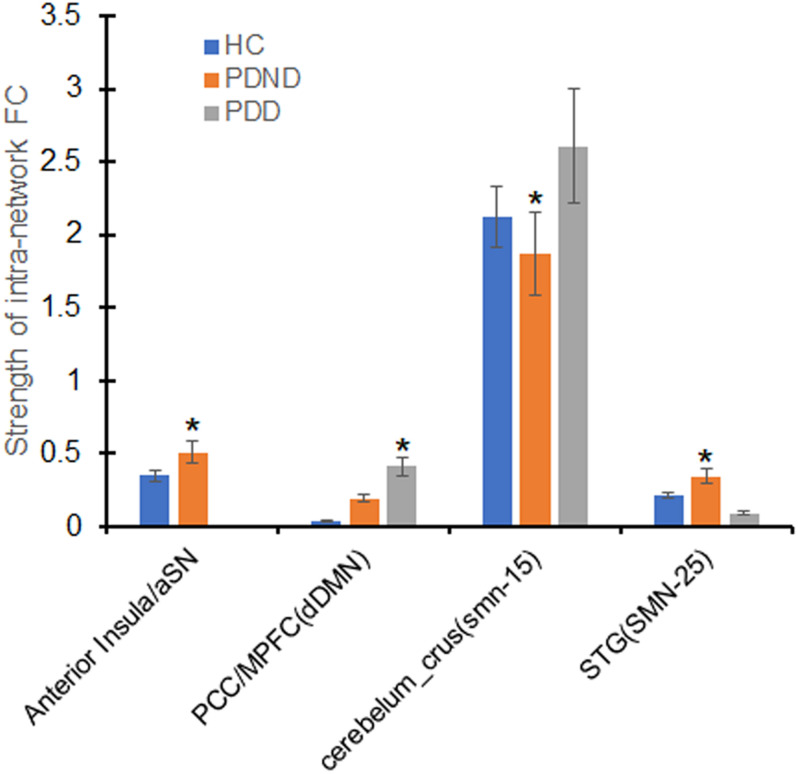
Differences of in the intra-network functional connectivity (FC) analysis. The x-axis represents the brain regions of the resting-state networks (RSNs) (parentheses), and the y-axis represents the mean value of intra-network FC strength. Error bars indicate the standard errors of the means. ^∗^*p* < 0.05, uncorrected, PDD *vs.* PDND. aSN, anterior salience network; SMN (IC15/IC25), sensorimotor network; dDMN, dorsal default mode network; STG, superior temporal gyrus; PDD, Parkinson’s disease with depression; PDND, PD patients without depression; HC, healthy control.

**FIGURE 3:**
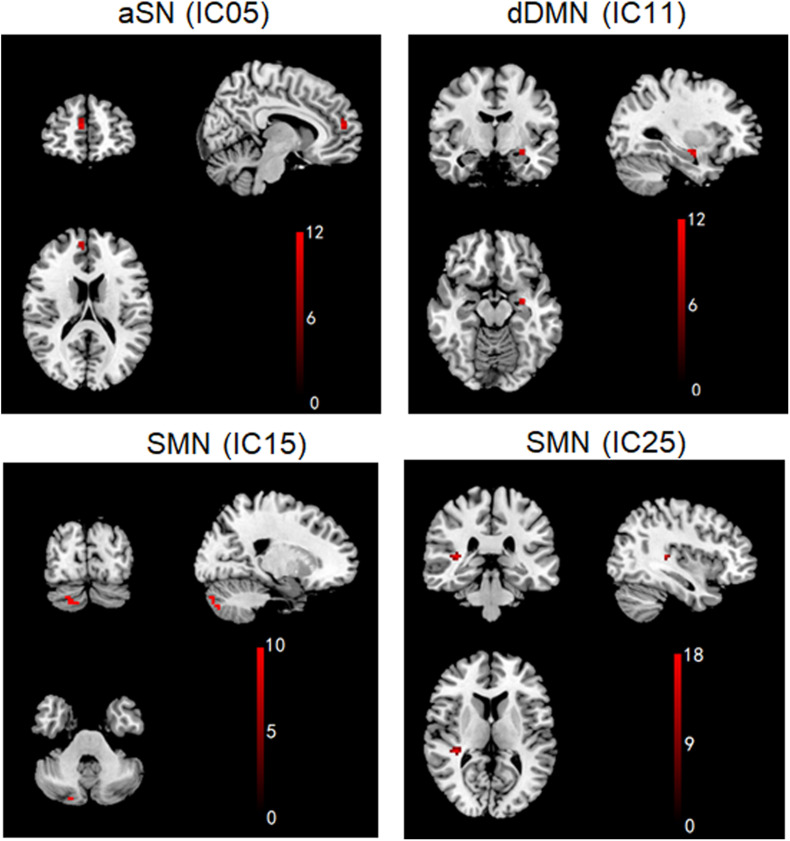
Brain regions with significant differences in intra-network functional connectivity (FC) between PDD and PDND patients. (*Post hoc* two-sample *t*-tests, *p* < 0.05, uncorrected, PDD *vs.* PDND). aSN (IC05), anterior salience network; IC, independent component; dDMN (IC11), dorsal default mode network; SMN (IC15/IC25), sensorimotor network.

aSN, dDMN, and SMN exhibited significant changes in FC among the PDD, PDND, and HC groups (ANOVA, *p* < 0.05, Figure 2). Compared with the HC group, the PDD and PDND patients showed increased connectivity in the left hippocampus of the dDMN (Hippocampus_L). The PDD patients exhibited an increased connectivity in the left hippocampus of the dDMN (Hippocampus_L) and the right Cerebelum_Crus2 of the SMN (Cerebelum_Crus2_R) and a decreased connectivity in the right medial superior frontal gyrus (mSFG) of the aSN (Frontal_Sup_Medial_R) and the right superior temporal gyrus (STG) of the SMN (Temporal_Sup_R) than did the PDND group (*p* < 0.05, Figure 3 and Table 2). No group differences in intra-network connectivity of the vDMN, PCUN, left CEN (LCEN), and right CEN (RCEN) were observed.

**TABLE 2:** Brain regions with significant differences in intra-network functional connectivity (FC) between PDD and PDND patients.

RSN	Brain region (AAL)	Number of voxels (cluster size)	MNI coordinates of local maxima			Peak Z/T value
			X	Y	Z	
aSN (IC05)	Frontal_Sup_Medial_R	14	9	51	15	11.1233
dDMN (IC11)	Hippocampus_L	12	−*03*	−*6*	−*51*	11.2688
SMN (IC15)	Cerebelum_Crus2_R	18	24	−*48*	−*33*	9.4682
SMN (IC25)	Temporal_Sup_R	13	36	−*33*	9	16.6457

**Post hoc* two-sample *t*-tests, *p* < 0.05, uncorrected. PDD *vs.* PDND. aSN (IC05), anterior salience network; IC, independent component; dDMN (IC11), dorsal default mode network; SMN (IC15/IC25), sensorimotor network.*

### Inter-Network Connectivity Analysis

**FIGURE 4:**
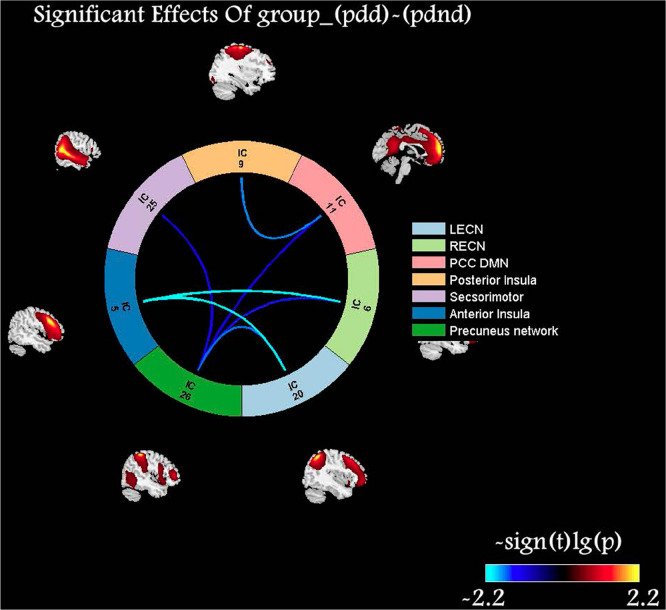
Comparisons of inter-network functional connectivity (FC) alterations in the resting-state networks (RSNs) between the Parkinson’s disease with depression (PDD) and PD without depression (PDND) groups. Color scale denotes the *t* value. Compared with PDND patients, PDD patients exhibited a decreased connectivity between anterior salience network (SN) (aSN) and bilateral executive control network (ECN), between posterior SN (pSN) and posterior default mode network (pDMN), and between precuneus network (PCUN) and pDMN/SMN/bilateral ECN.

The PDD patients exhibited a decreased connectivity between aSN and bilateral ECN, between pSN and dDMN, and between PCUN and dDMN/SMN/bilateral ECN than did PDND patients (Figure 4). The PDD patients exhibited decreased connectivity between aSN/pSN and dDMN and between PCUN and dDMN/vDMN/SMN/RECN than did the HCs. The PDND patients exhibited a decreased connectivity between aSN and pSN, and PCUN and vDMN, as well as an increased connectivity between aSN and RECN than did the HCs. These alterations indicated an impaired FC between SN/PCUN and the other networks in the PDD and PDND patients.

### Correlation Analysis of Functional Connectivity With Neuropsychological Test Scores and Clinical Characteristics

The Pearson correlation analysis demonstrated no significant correlations between altered FC values and any clinical variables (UPDRS-III/HDRS-17/BDI-21) in the patients with PD.

### Intra-Network Connectivity Predicts Depression Levels in Parkinson’s Disease Patients

After demographical and clinical variables were controlled for, including age, gender, educational years, illness duration, MMSE scores, and UPDRS-III scores, connectivity within the left hippocampus of dDMN and the right mSFG of aSN was a significant predictor of depression level in the PD patients, when these FCs were considered either simultaneously or separately in the regression model after correction (*p* < 0.05/4). Connectivity within the right Cerebelum_Crus2 of the SMN reached a level of *p* < 0.05, while it could not survive the correction (Table 3).

**TABLE 3:** Intra-network connectivity predicting depression levels in PD patients (*N* = 96).

Predictors	Variable statistics			Model statistics		
	β (s.e.)	*t*	*P*	Δ*R*^2^	*F*	*p*
**Step 1 (for all models)**						
Age	0.09 (0.06)	1.61	0.111	0.20	3.51	0.004
Gender	1.28 (1.14)	1.12	0.26			
Educational years	0.05 (0.17)	0.27	0.788			
UPDRS-III	0.21 (0.05)	4.39	<0.001			
MMSE	−0.04 (0.22)	−0.20	0.846			
Duration	−0.11 (0.28)	0.42	0.679			
**Step 2**						
Model 1^a^						
dDMN (Hippocampus_L)	5.04 (1.43)	3.53	0.001	0.23	6.91	<0.001
aSN (Frontal_Sup_Medial_R)	−2.57 (0.71)	−3.58	0.001			
SMN_15 (Cerebelum_Crus2_R)	1.16 (0.57)	2.02	0.047			
SMN_25 (Temporal_Sup_R)	–	–	–			
Model 2						
dDMN (Hippocampus_L)	5.58 (1.55)	3.61	0.001	0.11	3.51	0.004
Model 3						
aSN (Frontal_Sup_Medial_R)	−2.78 (0.78)	−3.56	0.001	0.10	5.22	<0.001
Model 4						
SMN_15 (Cerebelum_Crus2_R)	1.58 (0.64)	2.45	0.016	0.05	4.04	0.001
Model 5						
SMN_25 (Temporal_Sup_R)	−2.48 (1.37)	−1.81	0.073	0.03	3.51	0.002

*^*a*^Predictors included intra-connectivity of dDMN, aSN, SMN_15, and SMN_25. Using the stepwise method, connectivity of dDMN, aSN, and SMN_15 was significant and retained in the final model. SMN_15 could not survive the Bonferroni correction (*p* < 0.05/4). UPDRS-III, Unified Parkinson’s Disease Rating Scale-motor part III; MMSE, Mini-Mental State Examination; aSN (Frontal_Sup_Medial_R), anterior salience network (the right medial superior frontal gyrus); dDMN (Hippocampus_L), dorsal default mode network (the left hippocampus); SMN_15 (Cerebelum_Crus2_R), sensorimotor network_15 (the right Cerebelum_Crus2); SMN_25 (Temporal_Sup_R), sensorimotor network_25 (the right superior temporal gyrus).*

## Discussion

The present study systematically investigated the connectivity changes within and between PCUN, SMN, and the triple-network in the PDD and PDND patients. Compared with the PDND patients, the PDD patients showed a decreased intra-connectivity in the aSN and SMN; increased intra-connectivity in the dDMN and SMN; and decreased inter-connectivity between aSN and bilateral ECN, between pSN and dDMN, and between PCUN and dDMN/vDMN/SMN/RECN. Our study provided evidence of aberrant connectivity patterns across broad-scale networks in PDD.

In this study, we found that the PDD patients showed decreased inter-connectivity between aSN and bilateral ECN, and between pSN and dDMN. The triple-network model supports cognitive, affective, perceptual, and social functions (Menon, 2011). Thus, the disconnected communication from SN to ECN/DMN may cause disrupted top-down cognitive modulation over the limbic and subcortical structures, which may be involved in the onset and maintenance of depression. In addition, the occurrence of depression might arise from disturbance in more distributed neural networks, such as functional disruption of the prefrontal–limbic network (Luo et al., 2014). Consistently, previous studies also demonstrated that the SN is involved in PDD. For instance, Huang et al. (2020) reported that the disrupted connection between the SN and ECN might contribute to depression in PD. Wei et al. (2017) reported that the involvement of BGN, DMN, LFPN, and SN in PD facilitates the development of depression. Therefore, the impaired interactions between the triple network are critical for the occurrence and maintenance of depression in PD.

In this study, the PDD patients exhibited decreased inter-network connectivity between precuneus and DMN/SMN/ECN. Previous studies have demonstrated that the abnormal function of the striatum and the associated limbic–BGN circuitry is proposed to play a role in the emotional processing system in patients with depression (Jiao et al., 2011; Marchand et al., 2012; Andreescu et al., 2013). Studies have also shown that SMN is activated during bimanual motor tasks, suggesting the involvement of SMN in a pre-mediated state that prompts the brain in performing/coordinating a motor task (Beckmann et al., 2005). Thus, the vulnerability of the distinct PCUN and other large-scale networks plays a major role in the pathophysiology of neurodegenerative processes in the PDD patients. Our results suggest that the functional integrity of prefrontal–limbic–striatal–cortex circuitry function is involved in the pathogenesis of PDD.

In this study, the PDD patients had decreased connectivity in the right STG of the aSN and the right mSFG of the SMN and increased connectivity in the left hippocampus of the dDMN and the right Cerebelum_Crus2 of the SMN. Previous studies have shown hypoactivity, decreased metabolism, reduced density, and FC of the STG in PDD patients (Feldmann et al., 2008; Ballanger et al., 2012; Lou et al., 2015). The prefrontal cortex containing the mSFG serves an important function as a top-down modulator of emotional tasks (Phillips et al., 2003). The Sheng et al. (2014) study with ReHo and FC methods found that PDD patients had increased regional activity in the left frontal and medial frontal gyri than had non-depressed PD patients. Our previous study found abnormal ReHo values in both the cingulate cortex and the orbitofrontal area of depressed PD patients (Wang et al., 2020). Zhi et al. (2019) found that the right medial frontal gyrus activation could be a biomarker for the occurrence and the severity of depression in PD. The hippocampus is a major culprit of emotional dysregulation, contributing to the pathophysiology of depression in PD (van Mierlo et al., 2015). However, a previous study reported that PDD patients reduced connectivity of putamen with mesolimbic regions, particularly in the hippocampus (Luo et al., 2014). This discrepancy might be due to the neural network remodeling caused by anti-PD or anti-depressant medications. A meta-analysis study demonstrated that the Cerebelum_Crusl and Cerebelum_Crus2 areas were involved in executive functions and complex cognitive functions (including language, working memory, etc.) (Stoodley and Schmahmann, 2009). Both structural (Stoessl et al., 2011) and functional (Mentis et al., 2002) abnormalities of the cerebellum are present in movement and emotional disorders in depressed PD patients. Song et al. (2015) observed the interactive effects of motor and depressive symptoms on the bilateral posterior cerebellum.

Although some previous authors associated hyper-connectivity with functional compensation (Wang et al., 2006; Hillary et al., 2015), many others have considered increases in connectivity as a reflection of functional disruption (Esposito et al., 2010) and altered neuronal communication (Licata et al., 2013). It is therefore considered that “functional disconnection” implies both functional hypo-connectivity and hyper-connectivity. Recently, growing evidence (Küper et al., 2011; Song et al., 2015) has shown that aside from its traditional integration of motor functions, the cerebellum takes part in the regulation of non-motor functions, such as language, cognition, and emotion. Wen et al. (2013) observed an increased ALFF in the right cerebellum posterior lobe in depressed PD patients when compared with non-depressed PD patients. The increases in connectivity in the right cerebellum might be not only a compensatory mechanism for the defective motor system but also a reflection of functional disruption and altered neuronal communication. Thus, the STG, mSFG, hippocampus, and Cerebelum_Crus2 are likely the critical hubs in the neural circuits related with depression in PD patients.

Several limitations should be considered when interpreting the results of the present study. First, although the adoption of a 38-component ICA model was auto-estimated, there was no clear computational criterion for the number. Second, no significant correlations were found between the variables (intra-FC and inter-FC) and motor or non-motor scale scores (UPDRS-III/HDRS-17/BDI-21). This may be biased due to the small sample size in PDD group. Further studies in a larger sample size will be necessary to explore the relationship between connectivity abnormalities and the severity of depression in PDD. Third, it should be noted that none of our results could survive at the strict AlphaSim correction level of *p* < 0.001. However, in this study, the combination of the AlphaSim correction of *p* < 0.05 with individual voxel threshold at *p* < 0.001 and the required cluster size adopted was acceptable based on the previous studies (Kong et al., 2013; Fan et al., 2017). Our power analysis also indicated reliable effect sizes for our results. We were cautious in generalizing our results, and further studies are needed. Fourth, the patients in the current study were not drug naïve. Although the anti-PD or anti-depressant medications were stopped for 12 h before imaging and neuropsychological testing, the potentially confounding effects of chronic medications could not be avoided. Fifth, the lack of a primary depression group left the question of whether PDD shares a common neurobiological substrate with primary depression unanswered. Finally, this is a cross-sectional study. It remains unsolved whether the found alterations represent state or trait characteristics of our patient cohort.

In conclusion, abnormal FC between RSNs may reflect the neuroimaging characteristics of the prefrontal–limbic–striatal–cortex circuitry-related networks. The RSNs in PDD or PDND patients are not restricted to the triple-network model but rather spread to other neural networks such as PCUN and SMN. Specifically, we found that both the aberrant intra- and inter-network FC is involved in several important hubs in the large-scale networks. This study highlighted that aberrant intra-network FC and inter-network FC can be biomarkers for distinguishing PDD from PDND, and our findings can help understand the neural foundation of the depression in PD.

## Data Availability Statement

The original contributions presented in the study are included in the article/supplementary material, further inquiries can be directed to the corresponding author/s.

## Ethics Statement

The studies involving human participants were reviewed and approved by the Medical Research Ethical Committee of the Second Xiangya Hospital. The patients/participants provided their written informed consent to participate in this study.

## Author Contributions

CT and XZ contributed to the conception and design of the study. HL, SC, QS, TW, YZ, ZM, TZ, and WS contributed to the data collection. HL and JF contributed to the data analysis. HL contributed to writing the manuscript. JL and JY contributed to the English-language revision. All authors contributed to the article and approved the submitted version.

## Conflict of Interest

The authors declare that the research was conducted in the absence of any commercial or financial relationships that could be construed as a potential conflict of interest.

## Footnotes

1 https://www.nitrc.org/projects/gift, version 2.0a.
